# Human rights in patient care: a special collection

**DOI:** 10.1186/s40985-020-00144-3

**Published:** 2020-12-21

**Authors:** Tamar Ezer, Judy Overall

**Affiliations:** 1 Human Rights Clinic, University of Miami School of Law, Miami, FL USA; 2 ASPHER Human Rights in Patient Care Initiative, Brussels, Belgium

## Abstract

This piece introduces the special Public Health Reviews collection on human rights in patient care (HRPC). Work on HRPC dates back to 2007 and an Open Society Foundations initiative in collaboration with partners in Eastern Europe and Central Asia. We found that for marginalized groups, health care settings often were places of coercion, punishment, and/or violence rather than of treatment or care. At the same time, health care providers often did not know of their legal obligations and how to incorporate human rights norms in their work. They themselves faced a lack of independence, unsafe working conditions, and sanctions for providing evidence-based care. Laws existed that could potentially address violations, but they were rarely enforced, and most people did not know what they were. HRPC brings human rights principles to health care delivery and addresses the rights of both patients and health care providers. It seeks to translate laws and procedures protecting rights into practical terms, linking national, regional, and international frameworks. The special collection explores various aspects of HRPC, including state responsibility in private health facilities, reproductive health, palliative care, and intersections with public health. It further explores dimensions relevant to particular populations, including Roma, people who use drugs, and transgender persons.

We are excited to introduce this special *Public Health Reviews* collection on human rights in patient care (HRPC). Work on HRPC dates back to 2007 and an Open Society Foundations initiative in collaboration with partners in Eastern Europe and Central Asia. We found that for marginalized groups, health care settings often were places of coercion, punishment, and/or violence rather than of treatment or care. For example, a partner in Kyrgyzstan who formerly used drugs recounted a torturous shoulder operation without any anesthesia as doctors hammered nails into his bones. When the patient asked why he was made to suffer, the doctor responded, “Because you are a drug user. If I give you anesthesia, you will remember your drugs and tomorrow go buy more” [1]. The patient told us that since he stopped using drugs, he was no longer afraid of the police, but he was still afraid doctors. Examples of other encountered abuses included segregation of Roma women in separate maternity wards, disclosure of HIV status, and forced sterilization of women with disabilities. At the same time, health care providers often did not know of their legal obligations and how to incorporate human rights norms in their work. They themselves faced a lack of independence, unsafe working conditions, and sanctions for providing evidence-based care. While there was need for law reform, the biggest gap was in implementation. Laws existed that could potentially address violations, but they were rarely enforced, and most people did not know what they were.

In an effort to address the rights of both patients and health care providers and bring human rights principles to health care delivery, we coined the concept of “human rights in patient care.” Unlike “patients’ rights,” this concept is rooted in inherent human dignity rather than consumer transactions and allows for limitations, enabling an analysis of competing claims. It recognizes the interrelation between patient and provider rights and contexts of “dual loyalty,” where providers face simultaneous obligations to patients and another party, often the state. It is also complementary to bioethics, bringing a focus on systemic issues and the role of the state, procedures for accountability and enforcement, and a critical role for advocacy and community mobilization [2]. HRPC is not separate from the health and human rights approach, but rather probes and develops one particular aspect of it.

HRPC projects seek to translate laws and procedures protecting rights into practical terms for legal practitioners, health care providers, and patients, linking national, regional, and international frameworks. Initial work focused on the production of resources. This included *Practitioner Guides* for lawyers interested in taking HRPC cases, developed by interdisciplinary country teams of lawyers, doctors, and other professionals, as well as international experts. The *Practitioner Guides* are detailed, practical, how-to manuals, covering both litigation and alternative mechanisms, such as ombudspersons and medical licensing bodies and advocacy with United Nations bodies, which examine both patient and provider rights and responsibilities and procedures for protection at national, regional, and international levels. Health care providers have further used the *Practitioner Guides* to obtain a firmer understanding of the legal basis for patient and provider rights and available mechanisms for enforcement [3]. Complementing the *Practitioner Guides*, advocates produced patient-friendly materials in collaboration with community partners, which provide basic information on rights and remedies tailored to particular marginalized populations [4]. Additionally, a chapter in the *Health and Human Rights Resource Guide*, hosted by the François-Xavier Bagnoud Center at Harvard University, provides an introduction to the field of HRPC [5]. These resources then served as a basis for education, trainings, litigation, and advocacy.

Engagement in scholarship has provided an opportunity for more rigorous exploration of the HRPC concept and its application. In 2013, Harvard University published a themed section on HRPC in its *Health and Human Rights Journal* [6]. The introductory article in this issue laid out the HRPC framework and provided a typology of core human rights applicable to patients and providers. It further differentiated the HRPC concept from other paradigms commonly applied to health care settings, such as the right to health (which overlaps with HRPC), patients’ rights, patient safety, and bioethics [2]. Other articles in that issue reflect on work in the field, including education [7], litigation [8], and legislative gaps [9–11]. This special collection published by *Public Health Reviews* deepens this scholarship and reflects the geographic expansion of this work beyond Eastern Europe and Central Asia. The special collection explores various aspects of HRPC, including state responsibility in private health facilities, reproductive health, palliative care, and intersections with public health. It further explores dimensions relevant to particular populations, including Roma, people who use drugs, and transgender persons.

In the preface, Dainius Pūras, former United Nations Special Rapporteur on the right of everyone to the enjoyment of the highest attainable standard of physical and mental health, reflects on his career as a doctor, specializing in child psychiatry and social pediatrics, and the powerful potential for human rights to strengthen the delivery of care. He points to several paradoxes in modern medicine, including the development of groundbreaking treatment but lack of care to the most vulnerable; treatment protocols denying access to psycho-social care, harm reduction, and safe abortion in contradiction to science and evidence; and violations of physical integrity and dignity in the name of medicine. He calls on medical professionals to lead rights-based reform in patient care [12].

In “Human Rights and the Practice of Medicine,” Joanna Erdman, the McBain Chair in Health Law and Policy at the Schulich School of Law at Dalhousie University in Canada, explores how human rights education for health professionals can lead to new knowledge, changes in behavior and culture, and social justice advocacy. She surveys various educational initiations, analyzing the different methods by which they engage with health professionals and highlighting the transformative potential of HRPC education. She points out, “[T]he content of international human rights law is dynamic, evolving over time through ongoing and collective interpretation. To understand the nature of law in this way opens up a new relationship between the health professions and the law. Rather than being merely subject to the law, they have a role to play in making the law. After all, it is ultimately health providers who will give true meaning to the abstract standards of human rights law through their interpretation in the daily practice of patient care” [13].

Ximena Andión Ibañez, Director of the Mexican Instituto de Liderazgo Simone de Beauvoir, and Tamar Dekanosidze, strategic litigation lawyer at the Georgian Young Lawyers’ Association and former HRPC Legal Fellow, examine recent decisions by the U.N. Committee on the Elimination of Discrimination Against Women and the European Court of Human Rights on the state’s obligation to regulate private health facilities. They situate these decisions within the wider debate on state obligations in private settings and probe their implications for standard development [14].

Priti Patel, former Deputy Director of the Southern African Litigation Centre, discusses the forced sterilization of marginalized women as discrimination. She points to shortcomings in recent court decisions in Namibia and Europe, which find forced sterilization to violate individual rights, but fail to acknowledge the discriminatory and systemic nature of this practice. She applies key principles in the HRPC framework and provides recommendations for addressing forced sterilization claims [15].

The article by Nesime Salioska, Director of Roma S.O.S. in the Republic of Northern Macedonia, in collaboration with Theodore T. Lee and Ryan Quinn, applies the HRPC framework to access to health insurance for Roma in Macedonia. It further looks at the role of the judiciary, legislature, and advocates in the realization of HRPC [16].

Gabriela Barros de Luca, Violeta Zopunyan (a former HRPC Legal Fellow), Naomi Burke-Shyne, Anahit Papikyan, and Davit Amiryan, staff of the Open Society Foundations and the Center for Rights Development in Armenia, further apply the HRPC framework to the sphere of palliative care, examining the rights of both patients and providers. Through a case study in Armenia, they delve into the role of advocacy in creating an enabling environment for palliative care and HRPC more broadly [17].

Mikhail Golichenko and Sandra Ka Hon Chu from the Canadian HIV/AIDS Legal Network apply the HRPC framework to drug treatment and punishment in Russia. They specifically focus on “narcologists,” or drug treatment doctors in Russia, and analyze how punitive drug policy manifests as a violation of the rights of narcologists, who may lose their professional independence and ability to work according to professional standards and ethical norms [18].

Maya Peled-Raz, of the University of Haifa, Israel, School of Public Health and International Center for Health, Law and Ethics, contends that HRPC is a conceptual link between medical law with its patients’ rights subpart and public health law. She notes that while interrelated and complimentary, both are limited in effect and scope and neither sufficiently provides a remedy for systematic mistreatment in the health care system. She explains via analysis and a case study that HRPC can serve as a gap-building approach to the promotion of both individual and community health, applying human rights discourse and law to the patient care setting, while moving away from the narrow consumeristic view of health care delivery [19].

In the latest article in the collection, Amets Suess Schwend, of the Andalusian School of Public Health, Spain, analyzes the shared focus and potential alliances between the transgender depathologization perspective and the HRPC framework. The author explains the exposure of trans persons to multiple human rights violations in clinical practice and research and suggests a relevant strategy of collaboration among trans activists, scholars, and health professionals in developing a new trans health care model based upon a depathologization and human rights perspective, with the HRPC framework as a entry point for shared knowledge production [20].

We hope this collection of articles will serve as a starting point to ensure health care settings become true places of care everywhere and for everyone.

## Data Availability

Not applicable.
